# Sarcopenia discriminates poor prognosis in elderly patients following emergency surgery for perforation panperitonitis

**DOI:** 10.1002/ags3.12281

**Published:** 2019-08-16

**Authors:** Nobuhide Kubo, Hirohumi Kawanaka, Shoji Hiroshige, Hirotada Tajiri, Akinori Egashira, Hideya Takeuchi, Toshifumi Matsumoto, Eiji Oki, Tokujiro Yano

**Affiliations:** ^1^ Department of Surgery Beppu Medical Center National Hospital Organization Beppu Japan; ^2^ Clinical Research Institute Beppu Medical Center National Hospital Organization Beppu Japan; ^3^ Department of Surgery and Science Graduate School of Medical Sciences Kyushu University Fukuoka Japan

**Keywords:** complication, emergency surgery, mortality, panperitonitis, sarcopenia

## Abstract

**Aim:**

Sarcopenia has been reported as a prognostic predictor in various conditions; however, it has not been examined in patients with perforation panperitonitis.

**Methods:**

A total of 103 consecutive patients with perforation panperitonitis who underwent emergency surgery from 2008 to 2016 were retrospectively evaluated. Skeletal muscle index (SMI) was measured as the cross‐sectional area (cm^2^) of skeletal muscle in the L3 region on computed tomography images normalized for height (cm^2^/m^2^). Sarcopenia was defined as an SMI of ≤43.75 and ≤41.10 cm^2^/m^2^ in men and women, respectively. The impact of sarcopenia on postoperative outcomes was investigated.

**Results:**

Sarcopenia was present in 50 (48.5%) patients. Severe complications (Clavien‐Dindo grade ≥IIIb) and in‐hospital mortality were more frequently observed in patients with than without sarcopenia (28.0% vs 9.4%, *P* = .015) (20.0% vs 5.7%, *P* = .029) respectively. Multivariate analysis showed that age, sarcopenia, and renal dysfunction were independent risk factors for severe complications and in‐hospital mortality. The optimal cut‐off levels of age and SMI for predicting these were ≥79 years and SMI <38 cm^2^/m^2^, respectively. Among the patients aged ≥79 years, those with SMI <38 cm^2^/m^2^ had a severe complication rate of 71% and an in‐hospital mortality rate of 57%, whereas the rate of those with SMI ≥38 cm^2^/m^2^ was 22% (*P* = .011) and 11% (*P* = .008), respectively.

**Conclusion:**

Sarcopenia is a predictive factor of severe complications and in‐hospital mortality following emergency surgery for perforation panperitonitis, especially in elderly patients. Estimation of sarcopenia may identify patients eligible or not eligible for emergency surgery among elderly patients.

## Abstract 2

Sarcopenia is a predictive factor of severe complications and in‐hospital mortality following emergency surgery for perforation panperitonitis, especially in elderly patients. Estimation of sarcopenia may discriminate patients eligible or not eligible for emergency surgery among elderly patients.

## Graphical abstract

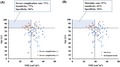

## 1 INTRODUCTION

Patients with panperitonitis caused by perforation of the digestive and biliary tract require emergency surgery, but their postoperative course is often difficult to manage. High rates of complications and hospital death are usually reported especially in elderly patients.^1^, ^2^ Several preoperative predictive systems, such as the Calculation of post‐Operative Risk in Emergency Surgery (CORES) score^3^ and Physiological and Operative Severity Score for the enUmeration of Mortality and morbidity (POSSUM),^4^ have been reported to assess morbidity and mortality in emergency surgery. However, these scoring systems require many variables, including subjective variables such as the American Society of Anesthesiologists (ASA) score, the Japan Coma Scale, or the Glasgow Coma Scale, and are time‐consuming. Moreover, these do not reflect the patient's general health status before admission.

In 1989, the term “sarcopenia” was proposed by Irwin Rosenberg to describe the age‐related decline of muscle mass.^5^ Sarcopenia is characterized by progressive and generalized loss of skeletal muscle mass and decreased skeletal muscle strength.^6^ Sarcopenia is a topic of high interest, and several studies have proven that sarcopenia is an indicator of a patient's general health status, such as nutritional and metabolic condition.^7^, ^8^, ^9^, ^10^ Although sarcopenia is reportedly a prognostic factor in various conditions such as chronic disease and metabolic disease,^11^, ^12^, ^13^, ^14^, ^15^, ^16^, ^17^ it has not been examined in patients with perforation panperitonitis.

In the present study, we evaluated the presence or absence of sarcopenia simply and objectively with the skeletal muscle index (SMI), indicated by the cross‐sectional area (cm^2^) of skeletal muscle in the third lumbar vertebra (L3) region on computed tomography (CT) normalized for height (cm^2^/m^2^).^18^ We herein aimed to elucidate the impact of sarcopenia on postoperative complications and mortality in patients with perforation panperitonitis following emergency surgery.

## 2 METHODS

### 2.1 Patients

A total of 103 consecutive patients with perforation panperitonitis who underwent emergency surgery from January 2008 to December 2016 at the National Hospital Organization, Beppu Medical Center were retrospectively reviewed. The definition of panperitonitis is contaminated ascites in the entire abdominal cavity from upper abdomen to pelvic floor with confirmation of operative record. Patients were excluded from this analysis if they underwent elective operation, were <15 years of age, or had injuries caused by trauma. There was no malignancy in any case. All patients underwent preoperative CT within 1 day of their emergency surgery. A transverse CT image at L3 was assessed on each scan. Cross‐sectional areas (cm^2^) of skeletal muscle in the L3 region were measured by outlining them on the CT images. The cross‐sectional areas were then normalized for height (cm^2^/m^2^) to obtain the skeletal muscle index (SMI).^19^ Sarcopenia was defined as an SMI of ≤43.75 cm^2^/m^2^ for men and ≤41.10 cm^2^/m^2^ for women based on previously reported cutoff points.^20^ The cross‐sectional area of skeletal muscle in the L3 region was measured by two individual researchers (N.K. and S.H.), and no significant difference in the mean value was obtained between them. The software application for sarcopenia evaluation was SYNAPSE Chronical Outlook for Patient's Examination Ver. 2.4.1 (SYNAPSE Enterprise‐PACS, Fujifilm Medical, Tokyo, Japan). All preoperative complications were defined as disease with drug treatment. In particular, patients with renal dysfunction had low estimated glomerular filtration (eGFR; <60 mL/min/1.73 m²) and were diagnosed with chronic renal dysfunction by a nephrologist or physician before hospitalization.

### 2.2 Study design

The following variables were compared between the patients with and without sarcopenia: patient demographics, ASA score, site of perforation, duration of hospitalization, postoperative complications, and in‐hospital mortality. Postoperative complications were graded according to the Clavien‐Dindo classification.^21^ Postoperative complications were defined as grade ≥II complications. Severe postoperative complications were defined as grade ≥IIIb complications (those requiring surgical intervention). The predictive factors were examined with respect to severe complications and in‐hospital mortality.

This study was approved by the institutional review board of the National Hospital Organization, Beppu Medical Center (2015‐027). It was carried out in compliance with the Helsinki Declaration and the International Conference on Harmonization of Guidelines for Good Clinical Practice.

### 2.3 Statistical analysis

Groups were compared using the Wilcoxon rank‐sum test (Mann‐Whitney *U*‐test). Categorical data were compared using the chi‐squared test or Fisher's exact test. Univariate and multivariate logistic regression analyses were carried out to control for variables that may confound the effect of sarcopenia on severe postoperative complications and mortality, with all parameters having a *P* value <.050 on univariate analysis included in a multivariate model. Univariate and multivariate associations are reported as odds ratio (OR) with its 95% confidence interval (CI). Receiver operating characteristic (ROC) curves were used to determine or predict severe complications and in‐hospital mortality. Areas under the ROC curves (AUC) were compared, and the cutoff levels were determined at an optimized accuracy with equal weight given to sensitivity and specificity errors. A *P* value of <.050 was considered statistically significant. All statistical analyses were carried out using JMP Pro 12.0 (SAS Inc., Cary, NC, USA).

## 3 RESULTS

Of 103 patients, 50 (48.5%) had sarcopenia. Characteristics of patients with and without sarcopenia are shown in Table 1. Women had a significantly higher rate of sarcopenia than men. Patients with sarcopenia had higher C‐reactive protein (CRP) than those without sarcopenia. Other factors such as age, white blood cell count (WBC), ASA score, preoperative complications except diabetes mellitus, site of perforation, and hospital stay were not related to sarcopenia. Complications (Clavien‐Dindo grade ≥II), severe complications (Clavien‐Dindo grade ≥IIIb) and in‐hospital mortality were more frequently observed in patients with sarcopenia than in those without (68.0% vs 45.3%, *P* = .019; 28.0% vs 9.4%, *P* = .015; and 20.0% vs 5.7%, *P* = .029, respectively). Details of postoperative complications are shown in Table 2. There was no statistical difference between the groups.

**Table 1:** Characteristics of patients with and without sarcopenia

Demographics	With sarcopenia (n = 50)	Without sarcopenia (n = 53)	*P* value
Age (y)	67.6 ± 17.4	68.6 ± 15.8	.76
Gender			
Male	17 (34)	31 (58)	.013
Female	33 (66)	22 (42)	
Laboratory data			
WBC (/mL)	10 162 ± 5539	9086 ± 5878	.342
CRP (mg/dL)	14.7 ± 10.9	10.4 ± 10.7	.046
SMI (cm²/m²)	35.7 ± 4.1	49.4 ± 5.1	<.001
ASA score			
1	5 (10)	5 (9)	.207
2	14 (28)	8 (15)	
3	28 (56)	36 (68)	
4	3 (6)	4 (8)	
Preoperative complications			
Circulatory disease	17 (34)	18 (34)	1
Respiratory disease	5 (10)	7 (13)	.634
Liver disease	1 (2)	3 (6)	.304
Renal dysfunction	12 (24)	9 (17)	.378
Diabetes mellitus	0 (0)	7 (13)	.008
Collagen disease	6 (12)	2 (4)	.132
Site of perforation			
Stomach and duodenum	20 (40)	19 (36)	.797
Small bowel and appendix	12 (24)	14 (26)	.907
Colon and rectum	17 (34)	16 (30)	.806
Biliary tract	0 (0)	3 (6)	.079
Uterus abscess	1 (2)	1 (2)	1
Postoperative outcomes			
Hospital stay (days)	38.4 ± 33.3	36.4 ± 35.9	.78
Complication (C‐D grade **≥**II)	34 (68)	24 (45)	.019
Severe complication (C‐D grade **≥**IIIb)	14 (28)	5 (9)	.015
In‐hospital mortality	10 (20)	3 (6)	.029

Normally distributed variables are presented as mean ± SD. Values in parentheses are percentages.

ASA, American Society of Anesthesiologists; C‐D, Clavien‐Dindo classification; CRP, C‐reactive protein; SMI, skeletal muscle index; WBC, white blood cells.

**Table 2:** Details of postoperative complications in patients with and without sarcopenia

Factor	With sarcopenia (n = 50)	Without sarcopenia (n = 53)	*P* value^a^
Surgical site infection			
Superficial and deep incisional			
II	0	3	1
IIIa	7	4	
IIIb	0	1	
Organ space			
II	0	1	.087
IIIa	4	1	
IV	1	0	
V	2	0	
Anastomotic leakage			
II	1	0	1
IIIa	1	2	
IIIb	1	1	
V	1	1	
Ileus			
II	0	1	.496
IIIa	0	1	
Pneumonia			
II	2	2	.31
IIIa	1	1	
IV	1	0	
V	2	0	
Pleural effusion			
IIIa	1	0	.111
IV	1	0	
V	1	0	
Atelectasis			
IIIa	1	0	.485
Pneumothorax			
V	1	0	.485
SIADH			
II	0	1	1
Brain infarction			
II	1	0	.485
Deep venous thrombosis			
IV	2	0	.233
Intra‐abdominal bleeding			
IIIa	1	0	.485
Gastrointestinal bleeding			
IIIa	1	0	.61
IV	0	1	
V	1	0	
Gastrointestinal perforation			
V	1	0	.485
Enterocolitis			
II	0	2	.496
Cholecystitis			
II	0	1	1
Spondyloarthritis			
IIIa	1	0	.485
Candidemia			
II	0	2	1
V	1	0	
Septic shock			
IIIa	0	2	1
IV	2	1	
V	1	0	
DIC			
II	1	3	.716
IIIa	2	1	
V	0	1	
Cardiac failure			
II	1	1	1
Renal failure			
V	1	0	.485
Multiple organ failure			
IIIa	0	1	.428
IV	1	0	
V	3	1	

Complications were graded according to the Clavien‐Dindo classification. Some patients had two or more complications.

DIC, disseminated intravascular coagulation; SIADH, syndrome of inappropriate antidiuretic hormone.

a Postoperative complications (Clavien‐Dindo grade ≥II) were compared between the two groups with Fisher's exact test.

Univariate and multivariate analyses showed that severe complications were significantly associated with age (OR, 11.72; 95% CI, 2.30‐59.88; *P* = .003), sarcopenia (OR, 4.41; 95% CI, 1.27‐15.34; *P* = .020), and renal dysfunction (OR, 5.77; 95% CI, 1.61‐20.73; *P* = .007) (Table 3). In univariate analysis, significant prognostic factors for in‐hospital mortality were age, sarcopenia, circulatory disease, respiratory disease, and renal dysfunction. Multivariate analysis identified age (OR, 15.07; 95% CI, 1.49‐152.92; *P* = .022), sarcopenia (OR, 5.43; 95% CI, 1.08‐27.34; *P* = .040), and renal dysfunction (OR, 13.08; 95% CI, 2.56‐66.76; *P* = .002) as independent prognostic factors for in‐hospital mortality (Table 4).

**Table 3:** Factors associated with severe complications

	Univariate analysis			Multivariate analysis		
	OR	95% CI	*P* value	OR	95% CI	*P* value
Age	9.35	2.03‐43.03	<.001	11.72	2.30‐59.88	.003
Male	0.61	0.22‐1.71	.35			
WBC	0.8	0.29‐2.19	.664			
CRP	1.41	0.52‐3.83	.497			
Sarcopenia	3.73	1.23‐11.31	.015	4.41	1.27‐15.34	.02
ASA score	1.33	0.43‐4.06	.62			
Preoperative complications						
Circulatory disease	2.01	0.73‐5.53	.173			
Respiratory disease	2.53	0.68‐9.50	.157			
Liver disease	0	0‐0	.332			
Renal dysfunction	5.4	1.82‐16.03	.001	5.77	1.61‐20.73	.007
Diabetes mellitus	0	0‐0	.192			
Collagen disease	2.96	0.64‐13.67	.148			
Perforation site						
Stomach and duodenum	0.95	0.34‐2.66	.919			
Small bowel and appendix	0.5	0.13‐1.87	.301			
Colon and rectum	2.25	0.81‐6.22	.118			
Biliary tract	0	0‐0	.403			
Uterus abscess	0	0‐0	.497			

ASA, American Society of Anesthesiologists; CI, confidence interval; CRP, C‐reactive protein, OR, odds ratio; WBC, white blood cells.

**Table 4:** Factors associated with mortality

	Univariate analysis			Multivariate analysis		
	OR	95% CI	*P* value	OR	95% CI	*P* value
Age	12	1.50‐96.19	.004	15.07	1.49‐152.92	.022
Male	0.68	0.21‐2.25	.53			
WBC	1.39	0.43‐4.48	.575			
CRP	3.08	0.88‐10.75	.068			
Sarcopenia	4.67	1.07‐16.16	.029	5.43	1.08‐27.34	.04
ASA score	2.75	0.57‐13.21	.19			
Preoperative complications						
Circulatory disease	3.73	1.12‐12.46	.025	1.16	0.23‐5.88	.856
Respiratory disease	4.56	1.14‐18.17	.022	7.51	0.91‐61.76	.061
Liver disease	0	0‐0	.438			
Renal dysfunction	9.48	2.68‐33.49	<.001	13.08	2.56‐66.76	.002
Diabetes mellitus	0	0‐0	.298			
Collagen disease	2.55	0.46‐14.20	.272			
Perforation site						
Stomach and duodenum	1.03	0.31‐3.40	.962			
Small bowel and appendix	0.22	0.027‐1.75	.084			
Colon and rectum	2.87	0.88‐9.36	.081			
Biliary tract	0	0‐0	.504			
Uterus abscess	0	0‐0	.587			

ASA, American Society of Anesthesiologists; CI, confidence interval; CRP, C‐reactive protein, OR, odds ratio; WBC, white blood cells.

**Figure 1:**
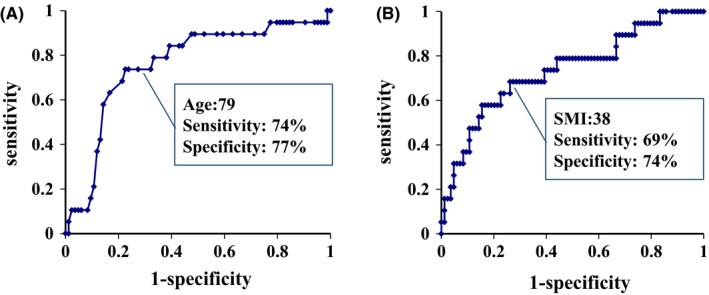
Receiver operating characteristic (ROC) analyses for prediction of severe complications with age and skeletal muscle index (SMI). A, ROC curve analysis shows that an optimal cutoff level of age is >79 y (sensitivity, 74%; specificity, 77%). Area under the ROC curve is 0.76. B, ROC curve analysis shows that an optimal cutoff level of SMI is <38 cm^2^/m^2^ (sensitivity, 69%; specificity, 74%). Area under the ROC curve is 0.74

**Figure 2:**
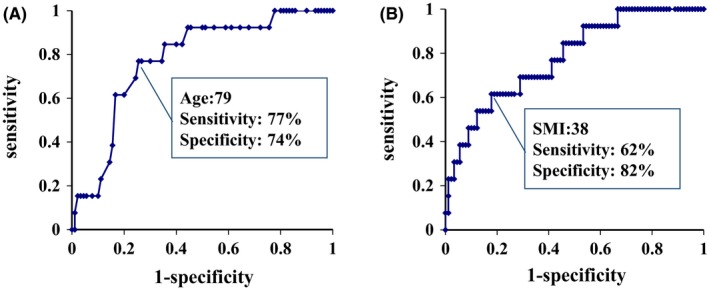
Receiver operating characteristic (ROC) analyses for prediction of in‐hospital mortality with age and skeletal muscle index (SMI). A, ROC curve analysis shows that an optimal cutoff level of age is >79 y (sensitivity, 77%; specificity, 74%). Area under the ROC curve is 0.78. B, ROC curve analysis shows that an optimal cutoff level of SMI is <38 cm^2^/m^2^ (sensitivity, 62%; specificity, 82%). Area under the ROC curve is 0.78

Receiver operating characteristic curves for predicting severe complications and optimal cutoff levels for age and sarcopenia were ≥79 years for age (sensitivity, 74%; specificity, 77%) and <38 cm^2^/m^2^ for SMI (sensitivity, 69%; specificity, 74%), respectively (Figure 1). ROC curves for predicting in‐hospital mortality and optimal cutoff levels for age and sarcopenia were ≥79 years (sensitivity, 77%; specificity, 74%) and <38 cm^2^/m^2^ for SMI (sensitivity, 62%; specificity, 82%), respectively (Figure 2).

**Figure 3:**
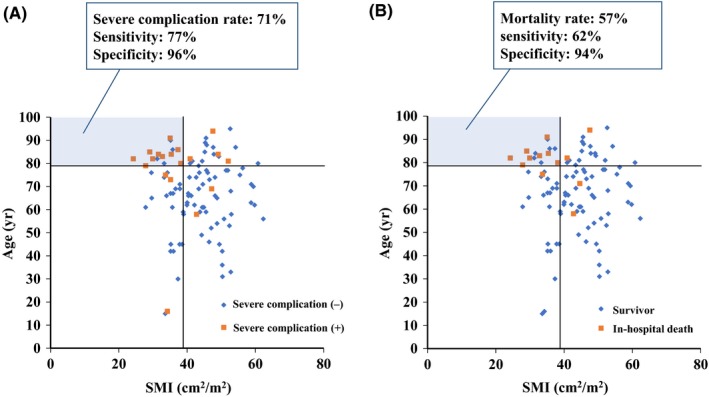
Distribution of age and skeletal muscle index (SMI) in patients with or without severe complications (A) and in‐hospital mortality (B). If age ≥79 y and SMI <38 cm^2^/m^2^ were used as the thresholds to predict severe complications (A) and in‐hospital mortality (B), the rate of severe complications was 71% (sensitivity, 77%; specificity, 96%) and the rate of in‐hospital mortality was 57% (sensitivity, 62%; specificity, 94%). Thresholds of 79 y and 38 cm^2^/m^2^ are indicated by solid lines

Distributions of age and SMI in patients with and without severe complications and mortality are indicated in Figure 3. If an age of ≥79 years and an SMI of <38 cm^2^/m^2^ were used as the thresholds to predict severe complications and in‐hospital mortality, the rate of severe complications was 71% (sensitivity, 77%; specificity, 96%) and the rate of in‐hospital mortality was 57% (sensitivity, 62%; specificity, 94%).

## 4 DISCUSSION

Sarcopenia, characterized by progressive and generalized loss of skeletal muscle mass and decreased skeletal muscle strength, have proven to represent the general health status of patients.^7^, ^8^, ^9^, ^10^ The European Working Group on Sarcopenia in Older People recommends defining sarcopenia by both loss of muscle volume and decreased muscle function.^6^ Although sarcopenia is associated with aging, it can also develop as a consequence of chronic disease or malignancy. For this reason, many definitions of sarcopenia have been established for various target cohorts such as ethnic groups and age groups.^22^ In the emergency setting, skeletal muscle function is difficult to evaluate and, thus, in the present study, muscle mass determined by CT images was measured. CT images, which are feasible in the emergency setting and completely objective, are usually carried out preoperatively for all patients with perforation panperitonitis. Because adjustment of skeletal muscle volume using body weight and body surface area^23^ is difficult in the emergency setting, we judged that using body height only was appropriate in the present study.

In the present study, sarcopenia was determined using SMI cutoff levels specific to each gender (≤43.75 and ≤41.10 cm^2^/m^2^ for men and women, respectively), based on a previous report with Japanese patients.^20^ Univariate and multivariate logistic regression models identified sarcopenia as an independent predictor of severe complications and in‐hospital mortality. When we examined the optimal cutoff of SMI for predicting severe complications and in‐hospital mortality, the optimal cutoffs of SMI were <38 cm^2^/m^2^ for both severe complications and mortality, regardless of gender. Using the common cutoff for both genders is simple and beneficial when preparing for emergency surgery. In patients with severe sarcopenia, as indicated by SMI <38 cm^2^/m^2^, the rates of complications, severe complications and in‐hospital mortality were 76%, 35% and 26%, respectively (Table S1).

Between 2000 and 2050, the world's population aged over 60 years will expand from 11% to 20%.^19^ Especially in Japan, the population aged over 65 years had already exceed 25% in 2015 and is expected to account for 38% of the population in 2050. Surgical procedures are thus being increasingly carried out in elderly patients. A prospective study with 4315 elective noncardiac procedures reported that elderly patients were at increasing risk of postoperative complications and in‐hospital mortality; the rates of postoperative complications and in‐hospital mortality in patients aged ≥80 years were 12.5% and 2.6%, respectively, and even in patients aged ≥80 years, in‐hospital mortality was low.^24^ A retrospective study with of 593 abdominal emergency surgeries showed that 30 day‐mortality and 90 day‐mortality were 10% and 17%, respectively, and that age was an independent risk factor of mortality.^25^ The present cohort with perforation panperitonitis indicated that age was an independent predictive factor of both severe complications and in‐hospital mortality and that patients aged ≥79 years, as an optimal cutoff, had severe complications of 43.8% and in‐hospital mortality of 31.3%. The age effect on morbidity and mortality may be more pronounced in emergency surgery, especially for perforation panperitonitis, than in elective surgery. In addition, because of the wide heterogeneity of general health status and the scarcity of objective tools for predicting operative risks, surgical decision‐making for emergency surgery is challenging in elderly patients. In the present study, among the patients aged ≥79 years, those with SMI <38 cm^2^/m^2^ had a severe complication rate of 71% and an in‐hospital mortality rate of 57%, whereas those with SMI ≥38 cm^2^/m^2^ had a severe complication rate of 22% (*P* = .011) and an in‐hospital mortality rate of 11% (*P* = .008). Among the patients aged <79 years, those with SMI <38 cm^2^/m^2^ had a severe complication rate of 15% and an in‐hospital mortality rate of 5%, and those with SMI ≥38 cm^2^/m^2^ had a severe complication rate of 4% (*P* = .132) and an in‐hospital mortality rate of 4% (*P* = 1.000). In a previous study of emergency abdominal surgery, sarcopenia significantly predicted mortality in univariate analysis but lost significance in multivariate analysis when factors such as age and ASA were included.^25^ In that study, the mean age was 61 years, but in the present study, it was 68 years and with 32 (33%) of the 103 patients aged ≥79 years. The impact of sarcopenia on surgical outcomes following emergency surgery may be less in younger patients than in elderly patients. Estimation of sarcopenia using SMI by CT images is thus likely to discriminate patients eligible or not eligible for emergency surgery among elderly patients with perforation panperitonitis.

Patients with sarcopenia had a higher incidence of complications and severe complications than those without sarcopenia. Postoperative complications observed in the present study may have been caused by a lack of multiple skeletal muscle functions. Skeletal muscle was recently identified as an endocrine organ and associated with immune regulation, glucose disposal, and protein synthesis.^26^, ^27^ Glutamine, an important nutrient for the integrity of the intestinal wall, is mainly synthesized in skeletal muscle. Sarcopenia is associated with reduced glutamine production, leading to intestinal dysfunction and infectious complications.^10^ Cytokine interleukin‐6 is secreted from skeletal muscle, promotes muscle hypertrophy, and enhances insulin‐stimulated glucose uptake. Sarcopenia may result in glucose intolerance because >75% of glucose is managed by skeletal muscle.^7^, ^8^, ^9^, ^10^ A high blood glucose concentration is associated with delayed wound healing and infectious complications.^28^, ^29^ These factors may be related to a higher tendency of surgical site infection of the organ space and pneumonia after emergency surgery in patients with sarcopenia, although there was no significant difference between the two groups in the present study. In addition, respiratory complications were found more frequently in 11 (50%) patients with sarcopenia compared with three (6%) patients without sarcopenia (*P* = .021). Sarcopenia has been reported as a risk factor for pneumonia because of poor chewing and swallowing function, delayed mobilization, dysphagia, or difficulty in clearing the airway.^30^

Although the present study has several limitations because of its retrospective design and small number of patients, we believe that sarcopenia is a predictive factor of severe complications and in‐hospital mortality following emergency surgery for perforation panperitonitis, especially in elderly patients. Estimation of sarcopenia using CT images and body height is simple and objective, and therefore convenient for the emergency setting. Rates of severe complications and in‐hospital mortality were extremely high in patients aged ≥79 years and with SMI <38 cm^2^/m^2^, but even in patients aged ≥79 years, those with SMI ≥38 cm^2^/m^2^ had better surgical outcomes. Estimation of sarcopenia is likely to identify patients eligible for emergency surgery among elderly patients with perforation panperitonitis. Evaluation of sarcopenia may identify patients susceptible to surgical stress and it may lead to the development of tailored preventive strategies and allocation of medical resources in the coming aging society.

## DISCLOSURE

Conflicts of Interest: Authors declare no conflicts of interest for this article.
