# Supplementary material for: Sarcopenia discriminates poor prognosis in elderly patients following emergency surgery for perforation panperitonitis
Source: Ann Gastroenterol Surg. 2019 Aug 16;3(6):630–7. doi: 10.1002/ags3.12281 (PMC6875939; doi:10.1002/ags3.12281)
Supplement: Supplementary file 1 [file AGS3-3-630-s001.docx]

| Demographics | Severe  sarcopenia (n=34) | Non-severe  Sarcopenia (n=69) | P value |
| --- | --- | --- | --- |
| Age (yr) | 67.6 ± 20.0 | 68.3 ± 14.7 | 0.830 |
| Gender  Male  Female | 7 (21)  27 (79) | 41 (59)  28 (41) | < 0.001 |
| Laboratory data  WBC (/μL)  CRP (mg/dL) | 10071 ± 5297  12.5 ± 9.9 | 9380 ± 5931  12.4 ± 11.6 | 0.566  0.989 |
| SMI (cm²/m²) | 33.7 ± 3.3 | 47.3 ± 6.0 | < 0.001 |
| ASA score  1  2  3  4 | 4 (12)  8 (24)  20 (58)  2 (6) | 6 (9)  14 (20)  44 (64)  5 (7) | 0.496 |
| Preoperative complication  Circulatory disease  Respiratory disease  Liver disease  Renal dysfunction  Diabetes mellitus  Collagen disease | 12 (35)  4 (12)  1 (3)  6 (18)  0 (0)  4 (12) | 23 (33)  8 (12)  3 (4)  15 (22)  7 (10)  4 (6) | 0.840  0.976  0.727  0.627  0.054  0.288 |
| The site of perforation  Stomach and duodenum  Small bowel and appendix  Colon and rectum  Biliary tract  Uterus abscess | 12 (35)  10 (29)  11 (32)  0 (0)  1 (3) | 27 (39)  16 (23)  22 (32)  3 (4)  1 (1) | 0.812  0.732  1.000  0.217  0.606 |
| Post-operative outcomes  Hospital stay (days)  Complication (C-D Grade ≥ II)  Severe complication  (C-D Grade ≥ IIIb)  In-hospital mortality | 40.5 ± 38.0  26 (76)  12 (35)  9 (26) | 35.8 ± 32.8  32 (46)  7 (10)  4 (6) | 0.520  0.004  0.002  0.019 |

Supplementary Table 1 Characteristics of patients with and without severe sarcopenia

Normally distributed variables are presented as mean ± standard deviation. Values in parentheses are percentages. ASA: American Society of Anesthesiologists, C-D: Clavien–Dindo classification, CRP: C-reactive protein, SMI: skeletal muscle index, WBC: white blood cells

C-D grades ≥ II complications were defined as complications.

C-D grade ≥ IIIb complications were defined as severe complications.
